# Decarbonising the Portland and Other Cements—Via Simultaneous Feedstock Recycling and Carbon Conversions Sans External Catalysts

**DOI:** 10.3390/polym13152462

**Published:** 2021-07-27

**Authors:** Sheila Devasahayam

**Affiliations:** Department of Chemical Engineering, Faculty of Science and Engineering, Monash University, Melbourne 3800, Australia; sheiladevasahayam@gmail.com

**Keywords:** cement decarbonization, waste utilization, co-pyro-gasification, carbon conversions, non-soot catalysts, clean energy

## Abstract

The current overarching global environmental crisis relates to high carbon footprint in cement production, waste plastic accumulation, and growing future energy demands. A simultaneous solution to the above crises was examined in this work. The present study focused on decarbonizing the calcination process of the cement making using waste plastics and biowastes as the reactants or the feedstock, to reduce the carbon footprint and to simultaneously convert it into clean energy, which were never reported before. Other studies reported the use of waste plastics and biowastes as fuel in cement kilns, applicable to the entire cement making process. Calcination of calcium carbonate and magnesium carbonate is the most emission intensive process in cement making in Portland cements and Novacem-like cements. In the Novacem process, which is based on magnesium oxide and magnesium carbonates systems, the carbon dioxide generated is recycled to carbonate magnesium silicates at elevated temperatures and pressures. The present study examined the Novacem-like cement system but in the presence of waste plastics and biomass during the calcination. The carbon dioxide and the methane produced during calcination were converted into syngas or hydrogen in Novacem-like cements. It was established that carbon dioxide and methane emissions were reduced by approximately 99% when plastics and biowastes were added as additives or feedstock during the calcination, which were converted into syngas and/or hydrogen. The reaction intermediates of calcination reactions (calcium carbonate–calcium oxide or magnesium carbonate–magnesium oxide systems) can facilitate the endothermic carbon conversion reactions to syngas or hydrogen acting as non-soot forming catalysts. The conventional catalysts used in carbon conversion reactions are expensive and susceptible to carbon fouling. Two criteria were established in this study: first, to reduce the carbon dioxide/methane emissions during calcination; second, to simultaneously convert the carbon dioxide and methane to hydrogen. Reduction and conversion of carbon dioxide and methane emissions were facilitated by co-gasification of plastics and bio-wastes.

## 1. Introduction

Current environmental challenges relate to meeting global CO_2_ emission targets, managing tons of plastics waste, and meeting the future energy needs. Cement production presents a major opportunity for addressing concerns related to waste plastics and biowastes as energy sources and chemical feedstock. This work identified and explored issues associated with this opportunity. These include:

Review of emission specifications and energy requirements for lime and clinker production

Comparative tonnage of present day energy sources vs. coal

Available plastics/energy sources

Feed-stock recycling of tires in cement production

Emission and toxicity concerns

Reactions of

o Conventional cement/clinkers/novel cements
o Cement/clinkers with plastics and/or biomass
o Cement/clinkers with plastics and biomass (co-pyro-gasification)
o Suppression of CO_2_ production
o Carbon conversions to hydrogen
o Non-soot forming catalytic calcines generated in situ

Issues obstructing commercialization

Recommendations for using ash from tires and silicones generated in situ as sand substitute.

### 1.1. Impetus for Decarbonizing Cement

Cement production accounts for the largest anthropogenic CO_2_ (~4 Gt/y) and ~8% of global CO_2_ emissions [1]. The current cement production rate greater than 4 Bt/y is set to increase to 23% by 2050 according to International Energy Agency (IEA). The current coal consumption rate for cement production is ~800 Mt/y (1 t cement production requires 200 kg coal; ~300–400 kg of cement is needed to produce 1 m^3^ concrete [2].

The cement sector is under pressure to reduce emissions by 16% per year before 2030 [3]. IEA’s Sustainable Development Scenario (SDS) aims to stay below 1.5 °C global warming [4] by adopting the following mitigation strategies: energy efficiency, alternate fuels, clinker replacement, state-of-the-art technologies (e.g., development of novel and carbon-negative cements), and carbon capture and storage [5,6]. Electrolytic production of lime from limestone as a strategy to cut down emissions was reported [5].

### 1.2. Novel Clinkers [7]

The SDS recommends reducing clinker to cement ratio by 0.64 before 2030 to reduce the emissions and the energy. Use of low carbon cements/novel clinkers can help reduce the ratio (Table 1) [8,9]. MgCO_3_ with similar chemistry to that of CaCO_3_ (except for the calcination temperatures) finds application in novel cements/clinkers; examples include carbon negative Novacem and eco-cement produced at ~700 °C and 750 °C, respectively, using fewer fossil fuels [10]. In comparison, Portland cement forms at 1450 °C, accompanied by higher emissions and energy consumption.

Engineers and contractors did not embrace these alternate cements due to high costs [11], and they prefer strong materials and strict building standards [9,12,13]. Geopolymer cement (USD 161.00) costs nearly thrice as much as the Portland cement (USD 51.00) [14,15]. High costs and lack of field testing prohibit the use of these new cements.

### 1.3. Simultaneous Decarbonization, Wastes Management, and Clean Energy Production in Portland Cements

The present study offers a novel step-change process to decarbonize the cements during the calcination via co-gasification of biomass and waste plastics, to cut down on emissions and energy requirement. In this section, energy requirement and source of emissions in cement making, roles of waste plastics and biowastes in reducing these emissions, and energy requirements are discussed.

#### 1.3.1. Emissions from Calcination of Carbonates, the Raw Materials Used in Cements

CaCO_3_, MgCO_3_, and dolomites are the raw materials used in cement making [16,17]. Calcination of calcite (limestone, CaCO_3_) requires higher energy than the calcination of dolomite and magnesite (MgCO_3_). The incipient evolutions of CO_2_ for magnesites, dolomites, and calcites occur at 640 °C, 730 °C, and 906 °C, respectively. In total, 1.092 kg CO_2_ is released per kg magnesia (MgO) and 0.477 kg CO_2_ per kg dolomite. The energy demand for MgO production ranges between 5 and 12 GJ/t MgO [18].

A cement clinker is made by calcining a homogeneous mixture of limestone (CaCO_3_) and clay or sand (silica and alumina source) in a rotary kiln at ~ 1450 °C (reaction 1) [9].

3CaCO_3_ + SiO_2_ → CaSiO_5_ + 3CO_2_ (1)

Subsequently, Portland cement is produced by grinding the clinker with ~5% of gypsum (calcium sulphate). There are two sources of CO_2_ emissions in Portland cement: (1) burning the coal as fuel and (2) calcination of limestone to lime. The focus of the present study was to reduce CO_2_ produced during the calcination of limestone to lime (reaction 2) as well as during the calcination of magnesite to MgO, a Novacem-like cement system.

About 65% of CO_2_ emissions are due to the calcination of raw materials, mainly from reaction 2. Energy consumption for lime (CaO) production is 4.25 GJ/t of quicklime [19]. The remaining 35% is due to fuel combustion. The amount of CO_2_ released is 1 kg/kg cement during calcination. Almost equal amounts of CO_2_ are released from heating up the required amount of coal. Coal consumption is 0.2 t/t cement. To produce 1 kg of clinker, 1.16 kg of limestone is required [20], of which CaO content is 0.65 kg/kg clinker. Emissions from 1 t clinker production are calculated as: 1 t × 65% × 0.79 = 0.51 t CO_2_ from CaCO_3_ calcination [21].

CaCO_3_ → CaO + CO_2_ (2)

#### 1.3.2. Role of Waste Plastics in Reducing the Emissions in Cement Processing

About ~104 Mt of waste plastics are projected to enter our environments by 2030 [22]. Wrong waste management practices of end-of-life (EOL) plastics pose huge environmental challenges. Plastics production and incineration will account for 56 Gg.t of carbon emissions between now and 2050 [23,24]. Halogenated and PVC plastics release dioxins, polychlorinated biphenyls, HBr, and furans into the environment. Harsh HCl gas from PVC can damage treatment plants and incinerators. Demand for silicones in electrical, electronics, medical, and other industries led to their increased land filling and the loss of valuable resources [25,26]. Toxic odors and severe temperatures constrain silicones repurposing. Net emissions factors for plastics for different materials management options are given in Table 2 [27,28].

The driving forces of plastics recycling schemes are energy recovery and cutting emissions, penalties, energy consumption, non-renewable resources, and manufacturing costs [29,30]. Energy recovery from waste plastics depends on their calorific values (kJ/kg): coke~25,000–30,000, PE~44,800, PP~42,700, PS~41,900, PET~23,200, PVC~1800, and epoxy (resin)~32,000 [31,32]. More than 90% of the plastics produced (300 Mt/y) are not recycled [33].

Industrial infrastructures such as coke ovens, blast furnaces, electric arc furnaces, and cement kilns provide alternative means for using waste plastics as fuels or as chemical feedstocks [29,30,34,35,36,37].

##### Waste Plastics as Fuel

A cement plant with 1 Mt capacity can consume between 10,000 and 30,000 Mt of plastics as fuel per year. About 50,000 t of waste plastics can be treated as fuel with 3000 to 4000 t of lime in a shaft kiln to generate syngas, which can support high temperature processes, such as glass foundries and iron and steel production replacing the fossil fuels [38,39].

##### Waste Plastics as Chemical Feedstock

**Figure 1:**
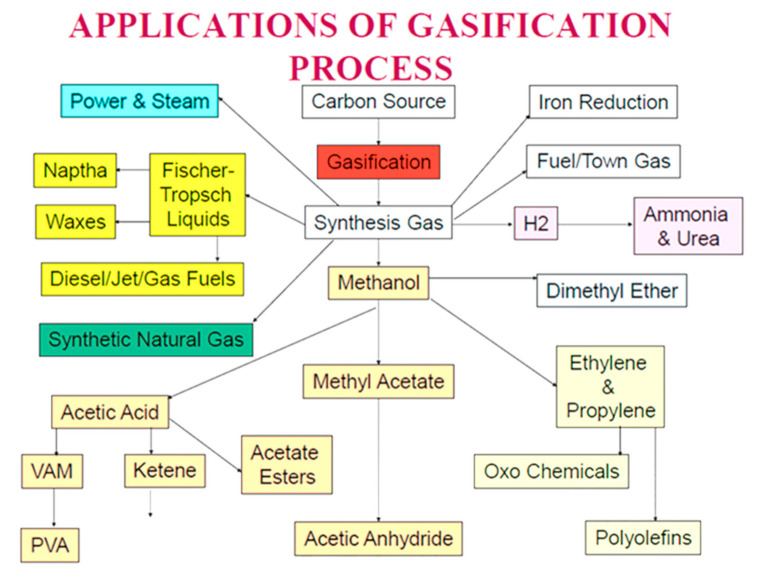
Multitude applications of syngas (Sengupta, 2020) (reproduced with permission).

Waste plastics are used as fuel in the cement industry but not as chemical feedstock (as raw material) to reduce the CO_2_ emissions thus far. Feedstock recycling of plastics is a sustainable solution to manage the plastic wastes such as mixed and halogenated plastic wastes and silicone wastes not suitable for recycling. Chemical feedstock recycling processes can extract valuable resources, e.g., C, H, Cl, and Si, from waste plastics, silicones, and biomass without considerable pre-treatment or depolymerization. During chemical feed stock recycling, plastics decompose without burning, producing chemically useful materials, and can convert to syngas at cement making temperatures [34,40]. Syngas is a renewable fuel with similar properties to natural gas that contains H_2_ and CO and has many applications, as seen in Figure 1 [41]. It is a precursor for liquid fuel production via Fischer–Tropsch process and a main source of H_2_ in the refineries [42].

Waste plastics as chemical feedstock in iron and steel industry reduce CO_2_ emissions, acting as reductants and as the source of syngas [29,30,31,43]. Up to 30% reduction in CO_2_ emissions is demonstrated in iron ore reduction using waste plastics as feedstock to partially replace coke as the reductant. Blast furnace and coke ovens treat waste plastics as chemical feedstock to produce syngases. The advantage of feedstock recycling in blast furnace approximates to 50 GJ/t of mixed plastics [44].

#### 1.3.3. Biomass/Biowastes

Biomass/biowastes are generally considered carbon neutral because the CO_2_ emitted to the atmosphere during combustion is absorbed while growing the replacement biomass. However, emissions accrue during farming, harvesting, processing, and delivering the fuel. A “carbon neutral” emissions factor for biomass is 0.04 kgCO_2_e/kWh (net CO_2_e emissions assuming carbon sequestration) and 0.39 kgCO_2_e/kWh when all emissions accrued at the point of consumption are considered [45]. The biomass has the following composition: cellulose 42%, lignin 29%, and hemicellulose 7% [46]. At high pyrolysis temperatures, biomass exhibits increased amounts of H_2_ and CO and decreased amounts of CO_2_ in the gases [47]. Carbon neutral natural rubber components in tires contribute to lower CO_2_ emissions [48].

#### 1.3.4. Decarbonizing Cement via Chemical Feedstock Recycling of Wastes

This study focused on decarbonizing the calcination phase (reaction 2) of Portland cement and Novacem-like cements and converting the CO_2_ generated during the calcination to hydrogen and/or syngas. This was achieved by calcining the mixture of plastic wastes and biowastes and the carbonates (calcium carbonate or magnesium carbonate).

The author’s previous studies formed the basis for the proposed decarbonization in Portland and Novacem-like cement processing. The author’s earlier studies similar to the Novacem process detail the low temperature and the high pressure carbonation (−13 °C and 6 bar) of silicate rich magnesites dumped as wastes and calcination reactions of MgCO_3_ to MgO producing CO_2_ [49,50,51,52,53]. Novacem production involves carbonation of magnesium silicates under elevated temperature and pressure (180° C/150 bar). The carbonates produced are heated up to 700 °C to produce MgO, where the CO_2_ generated is recycled back to carbonate the silicates [11].

Author’s earlier studies were extended by the author to incorporate plastics and biowaste during the calcination of MgCO_3_ to MgO, resulting in a great reduction in the carbon footprint and simultaneous production of hydrogen [54]. Author’s research on plastic degradation and use of plastics and forestry wastes in materials processing was the inspiration to extend the application of organic waste material to cement processing [29,30,55,56,57,58]. The author’s work on non-soot forming the catalytic ability of calcine intermediates, e.g., the MgCO_3_–MgO system in carbon conversion reactions, the dry reforming reaction to produce syngas and/or enriched hydrogen [59,60], underpins the application of waste organic materials in reducing the emissions in cement making.

The present study adopted the strategies discussed in the earlier works of the author, i.e., the use of organic wastes to decarbonize the calcination reactions of CaCO_3_ to CaO (reaction 2) of Portland cements and calcination reactions of MgCO_3_ to MgO of Novacem-like cements (“green” alternatives to Portland cement), which, to date, remain high carbon footprint processes. Similar chemistries between MgCO_3_–MgO and CaCO_3_–CaO systems enabled the author to extrapolate the results from one system to the other, accounting for slight differences in calcination temperatures and the amounts of CO_2_ released during the calcination reactions. The catalytic ability of these systems is exploited in carbon conversion reactions (dry reforming reactions) to produce syngas and hydrogen during the calcination phase. The authors’ study on replacing silica and coke with silicone wastes in ferrosilicone production formed the basis for recommending the use of silicone wastes in place of expensive silica in cement clinker production (reaction 1) [61].

## 2. Objectives

This study sought to resolve the high carbon footprint associated with Portland and Novacem-like cements and unsuitable plastic waste management strategies simultaneously. The CaCO_3_–CaO system of Portland cement and the MgCO_3_–MgO system of Novacem-like cements are reported with the overarching aim to minimize emissions, energy, and pollution. As the calcination phase (reaction 2) is the major emitter of global CO_2_, this study aimed to minimize the emission during this phase by introducing waste plastics and biowastes as chemical feedstock. This work did not focus on the use of plastic and bio wastes as fuel sources. Specific objectives included establishing the criteria for suppressing the CO_2_ (mainly from calcination of carbonates and gasification of wastes) and CH_4_ emissions (from co gasification of wastes) during the calcination and increasing the H_2_ generation during the calcination.

## 3. Experimental

The experiments involved the study of calcination reactions of CaCO_3_ (reaction 2) and MgCO_3_, responsible for the most emissions in cement making, in the presence of plastics and/or biomass. These experiments were designed to establish the criteria for emissions reduction and emission conversions to hydrogen, the source of green energy. The study included monitoring the off-gas composition from calcination experiments.

Materials used in this study included CaCO_3_, MgCO_3_-hydrate, epoxy resin (represented plastics), and *Pinus radiata* (represented biomass). Sigma Aldrich (M7179-500G) supplied CaCO_3_ and MgCO_3_-hydrate as anhydrous (MgCO_3_·xH_2_O, 40% to 44% Mg as MgO basis, molar mass 84.31). Huntsman Advanced Materials Pty Ltd. (Australia) supplied ARALDITE^®^ GY 191 CI Bisphenol A epoxy resin with the composition: bisphenol A epoxy resin greater than 60%; glycidyl ether of C12-C14 alcohols less than 30%; bisphenol F-epoxy resin less than 30%.

*Pinus radiata* was vacuum dried at 80 °C for 2 h and packed to a density of 400 kg m^–3^ in a furnace. The proximate and the ultimate analyses details of the *Pinus radiata* and the plastics are given in Table 3 [62,63].

Calcination experiments were carried out in a laboratory setup with furnaces [53,54,61]. Isothermal calcination of CaCO_3_ samples was carried out at 1250 °C and 1450 °C in an electricity operated horizontal tube furnace in an inert (argon) atmosphere at a flow rate of 1.0 L/min. The MgCO_3_ samples were subjected to isothermal calcination at 1000 °C in an IR image gold furnace and an arrangement of internals for heating. MgCO_3_ samples (20 to 50 mg) packed inside the silica tube were introduced at 1000 °C in the middle of a graphite heating element. Helium at ~50 mL/min was maintained.

### 3.1. Off-Gas Compositions

A gas chromatographic (GC) analyzer (SRI8610C Chromatograph Multiple Gas #3 GC) configuration equipped with a thermal conductivity conductor (TCD) and a continuous IR gas analyzer were used to measure off-gases, CH_4,_ and CO_2_ periodically for CaCO_3_ and CaCO_3_ + resin studies. The amounts of H_2_ could not be monitored during the calcination studies due to the limitation in the IR used.

The volatiles from the MgCO_3_ +resin +biomass system were measured with MTI Activon M200 series micro gas chromatograph (GC) instrument. The thermal conductivity detectors with a 5A molecular sieve column at 60 °C was used to measure H_2_ and CO. A Poraplot U column at 40 °C was used to measure CO_2_, CO, CH_4_, C_2_H_4,_ and C_2_H_6_. The evolution rate was determined as the wt.% of initial Wt. of sample/min.

### 3.2. X-ray Diffraction (XRD)

XRD with a copper Kα source operated at 45 kV and 40 mA and scanned at a step size of 0.026° and a scan rate of 1°/min and X’pert High score software were used for phase identification of calcined MgCO_3_ with epoxy resin at 1200 °C.

## 4. Results

### 4.1. Calcination of CaCO_3_

**Figure 2:**
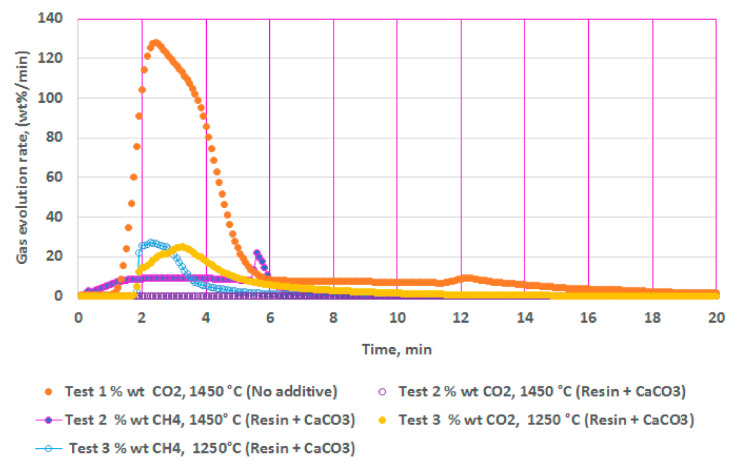
Gas chromatography results of isothermal calcination of: calcium carbonate (2.36 g) at 1450 °C; calcium carbonate (2.36 g) + resin (2.37g) at 1450 °C; and calcium carbonate (2.36 g) + resin (2.06 g) at 1250 °C.

Figure 2 illustrates the results from isothermal calcination reactions of CaCO_3_ with and without the plastic resin (resin). Figure 2 shows calcination of CaCO_3_ (2.36 g) at 1450 °C without the resin (test 1), calcination of CaCO_3_ (2.36 g) with the resin (2.37g) at 1450 °C (test 2), and calcination of CaCO_3_ (2.36 g) with the resin (2.06 g) at 1250 °C (test 3). Test 2 showed almost no traces of CO_2_ but only CH_4_, while test 3 showed reduced amounts of CO_2_ and almost equal amounts of CH_4_. These figures show the effects of the resin and the temperatures in suppressing the CO_2_ emissions to almost zero at high temperatures during the calcination. In these tests (tests 3 and 4), the resin quantity was kept almost equal or slightly less than the CaCO_3_ at 2.36 g.

To summarize, calcination of CaCO_3_ in test 2 showed more CH_4_ and negligible amounts of CO_2_ at 1450 °C; test 3 showed almost equal amounts of CO_2_ and CH_4_ at 1250 °C, demonstrating the effect of temperatures. Additionally, test 2 had slightly higher resin content than in test 3. Both the high temperature and the higher resin content could be responsible for suppressing the CO_2_ significantly. Hydrogen content was not measured during these tests. Biomass effect was not studied during calcination of CaCO_3_.

### 4.2. Calcination of MgCO_3_

**Figure 3:**
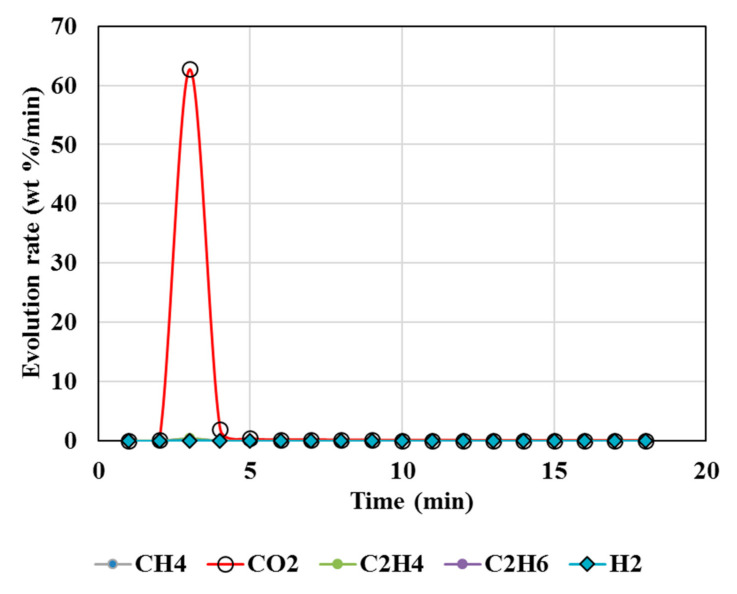
Gas Chromatography results of isothermal calcination of magnesite (Test 4, MgCO_3_·xH_2_O) at 1000 °C.

**Figure 4:**
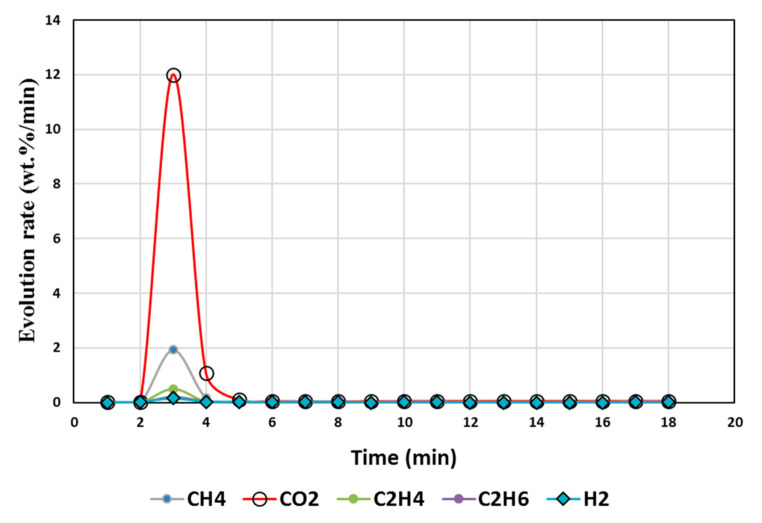
Gas Chromatography results of isothermal calcination of magnesite + biomass (Test 5, MgCO_3_·xH_2_O; biomass) at 1000 °C.

**Figure 5:**
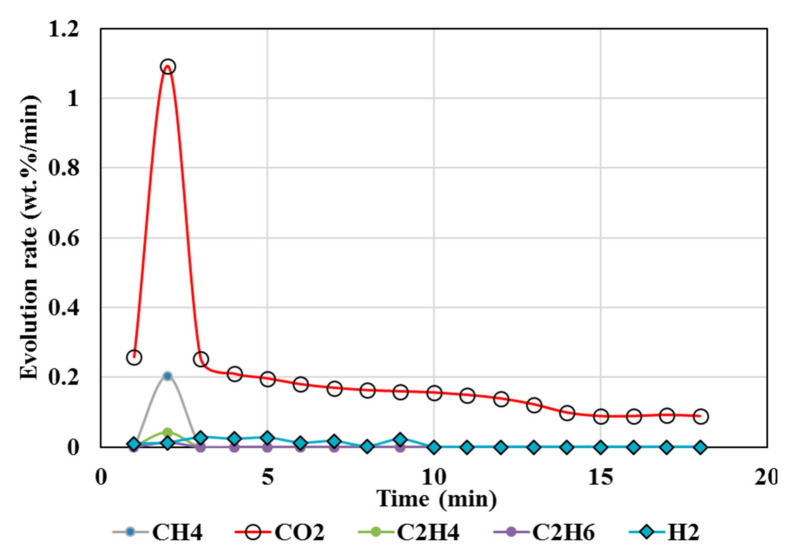
Gas Chromatography results of isothermal calcination of magnesite + plastics (Test 6, MgCO_3_·xH_2_O; resin) at 1000 °C.

**Figure 6:**
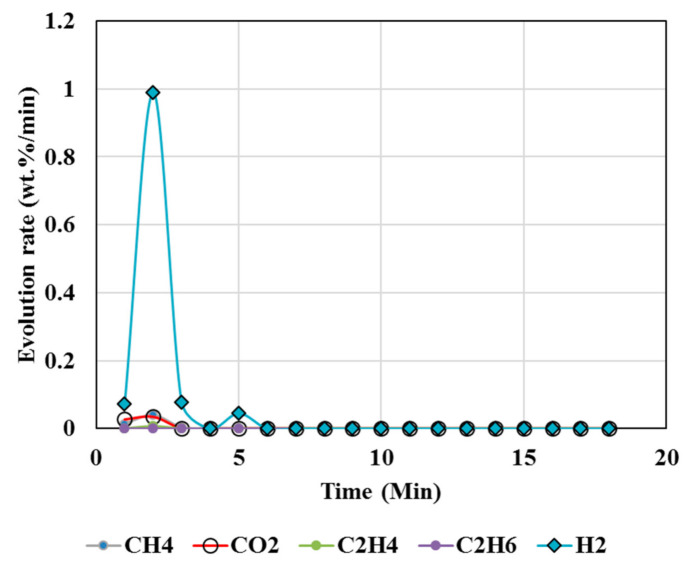
Gas Chromatography results of isothermal calcination of magnesite + plastics + biomass (Test 7, MgCO_3_·xH_2_O; biomass; resin) at 1000 °C.

**Figure 7:**
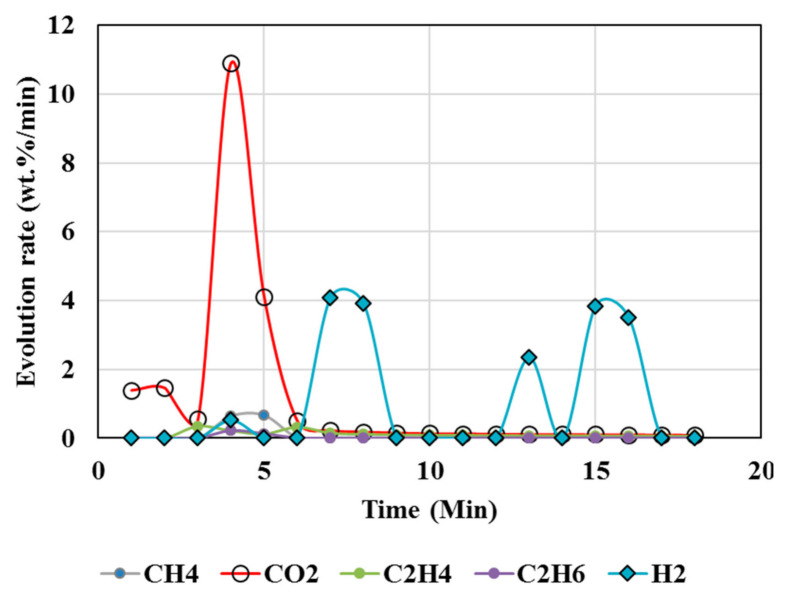
Gas Chromatography results of calcination of magnesite + resin + biomass (Test 8, MgCO_3_·H_2_O, biomass, resin) at 1000 °C.

Figure 3, Figure 4, Figure 5, Figure 6 and Figure 7 illustrate the results from isothermal calcination reactions of MgCO_3_ at different compositions of plastic resin (resin) and biomass. Figure 3 shows calcination of MgCO_3_ at 1000 °C without resin and biomass.

A summary of test details and results of calcination reactions of MgCO_3_·xH2O and CaCO_3_ with various ratios of biomass and plastics and at different temperatures is shown in Table 4, including the experimentally observed (y%) and the expected values for the gas evolution. The expected gas composition was calculated based on the mass% of different components (carbonates, biomass, and resin) present in each sample [54,60]. Cumulative gas compositions determined by GC are shown in Table 5.

High temperatures and high plastic (resin) content favored suppression of CO_2_ above 95%, as seen from the test results of tests 5, 6, 7, and 8 (Table 4 and Table 5), whereas biomass contributed to less suppression, e.g., up to 82% reduction in CO_2_ but up to 230% increase in hydrogen. A higher resin content than the biomass (test 7) during the calcination resulted in CO_2_ reduction up to 99%, and in CH_4_, there was a reduction up to 97% accompanied by 360% increase in H_2_ compared to the expected value. Test 7 could be an ideal scenario to produce H_2_ enriched gas. Resin content approximately equal to or less than the biomass during the calcination resulted in 76% reduction in CO_2_ and ~63% reduction in CH_4_ but 4684% increase in H_2_ compared to the expected value (Test 8).

## 5. Discussion

Note: biomass was not used in CaCO_3_ tests. During calcination of MgCO_3_·xH_2_O, the effect of temperature was not studied. Another interesting observation was the absence or the negligible amounts of CO, contrary to what was expected.

### 5.1. Low Carbon Portland Cement and Novacem-Like Cement

The calcination phase of the cement production is the most emission intensive process. Attempts to reduce CO_2_ emissions during the using waste plastics as the chemical feedstock were never reported before. Cement kilns use shredded waste plastics as fuel but not as chemical feedstock. The current study demonstrated the CO_2_ reductions during the calcination reactions of calcium carbonate (Portland cements) and magnesium carbonate (Novacem-like cements) in the presence of resin and/or biomass (Figure 2, Figure 4, Figure 5, Figure 6, Figure 7), indicating the feasibility of using cements/clinkers production as waste plastic conversion facilities. Owing to their similar chemistries, the results from MgCO_3_ studies can be extrapolated to CaCO_3_ (taking into consideration the higher calcination temperature of CaCO_3_ (906 °C), which is close to the iron ore reduction temperatures.

The role of plastics and biomass as feedstock in greatly reducing the carbon footprint of calcination reactions in cement making as well as the conversion of carbon from calcination reaction to syngas/hydrogen are explained in the following sections.

### 5.2. Chemical Feed Stock Recycling of Plastics

Plastics pyrolysis shows two phases, solid carbon and gas, namely CH_4_ and H_2_, which are thermodynamically stable at 1100 °C [64]. CH_4_ and the solid carbon further undergo catalytic transformation to syngases. The following reactions characterize the chemical feed stock recycling of waste plastics:

Plastics decomposition (pyrolysis) results in reaction 3:

Polymers → CnHm (g)(3)

Pyrolysis product from reaction 3 undergoes methane cracking (greater than 557 °C) (reaction 4):

C_n_H_m_ (g) → nC (s) + H_2_ (g); CH_4_ = C (s) + 2H_2_; ∆H = 75.6 kJ/mol (4)

Syngas production is governed by the following reactions:

The Boudouard reaction (reaction 5, ~701 °C):

C + CO_2_ → 2 CO; ∆H = 172 kJ/mol (5)

Water gas shift reaction (reaction 6):

CO + H_2_O (g) → CO_2_ + H_2_; ∆H = −41.2 kJ/mol (6)

Water gas reaction or char reforming (reaction 7, greater than 700 °C):

C + H_2_O → H_2_(g) + CO; ∆H = 131 kJ/mol (7)

Dry reforming reaction (~700° C in presence of catalysts) (reaction 8):

CH_4_ + CO_2_ → 2CO + 2H_2_; ∆H = 247 kJ/mol (8)

Methane reforming reaction (reaction 9)

CH_4_ + H_2_O → 3H_2_ + CO; ∆H = 206 kJ/mol (9)

Reactions 7 (water gas reaction), 8 (dry reforming reaction), and 9 (methane reforming reaction) result in various ratios of syngas. These reactions are endothermic and high temperature reactions requiring catalytic support. The main benefit of CO_2_ reforming methane (reaction 8, where CO_2_ acts as the oxidizing agent) is, when H_2_/CO is ~1, it is suitable for synthesizing oxygenated chemicals, e.g., methanol, acetic acid, aldehydes, ethanol, a wide variety of alcohols, olefins, and gasoline [65]. Oxygenates facilitate easy and safe storage and transport of energy. Methanol mixed with dimethyl ether (DME) is an excellent fuel for diesel engines with a high cetane number and beneficial combustion characteristics. The energy input for the CH_4_ dry forming reaction (reaction 8) is 20% higher than the steam reforming (or the methane reforming) reaction 9, resulting in syngas of varying H_2_/CO molar ratios. The drawback of the methane reforming (reaction 9) is that the H_2_/CO ratio 3:1 is greater than that required for the Fischer–Tropsch process.

### 5.3. Chemical Feedstock Recycling of Biomass

Biomass undergoes similar reactions as waste plastics during pyrolysis (refer to Section 5.2). Gases generated during the pyrolysis of biomass are CO, H_2_, CH_4_, and CO_2_; other products of pyrolysis include H_2_O and char depending upon the ambience [66]. Steam gasification/reduction chemical processes of biomass often occur at temperatures above 700 °C governed by: methane cracking (reaction 4), Boudouard (reaction 5), water gas shift (reaction 6), char reforming (reaction 7), dry reforming reaction (reaction 8), and methane reforming (reaction 9). During the gasification of biomass in an inert environment at 900 °C, cellulose contributes to CO, hemicellulose promotes CO_2_ generation, while lignin aids H_2_ and CH_4_ generation.

### 5.4. Reduction in CO_2_ Emissions during Calcination

Calcination of inorganic carbonates in reducing atmosphere (reactions 10 and 11) serves to capture or utilize CO_2_, the chemical H_2_ storage system for CH_4_, and the fuels from syngas [66,67,68,69,70]. Plastics and the biomass provide the reductive atmosphere to reduce the CO_2_ emissions during calcination. H_2_ produced in the reductive calcination can be a means to produce CH_4_ or CO/syngas from the CO_2_ emitted [71].

The methane cracking reaction (reaction 4) reduces the CO_2_ generated during calcination of MgCO_3_ and CaCO_3_ in the presence of plastic/biomass. Reduced CO_2_ emissions (reaction 10 and 12) in the presence of a reductive atmosphere of H_2_ and N_2_ mixtures was reported [71]. The H_2_ and the C, the products of reaction 4, react with MgCO_3_ (reactions 10 and 12) and CaCO_3_ (reactions 11 and 13), resulting in reduced CO_2_ emissions (reactions 10 and 11).

(a + b + c) MgCO_3_ + (b + 4c) H_2_ → (a + b + c) MgO + aCO_2_ + bCO + cCH_4_+ (b + 2c) H_2_O (10)

(a + b + c) CaCO_3_ + (b + 4c) H_2_ → (a + b + c) CaO + aCO_2_ + bCO + cCH_4_ + (b + 2c) H_2_O (11)

MgCO_3_ + C → MgO + 2CO (12)

CaCO_3_ + C → CaO + 2CO (13)

Calcination of magnesite in a reductive hydrogen atmosphere results in decreased CH_4_ and increased CO content. Amounts of CH_4_ formed in reactions 10 and 11 depend upon MgCO_3_ content, i.e., the amount decreases as MgCO_3_ content decreases. The CO increases as the MgO content increases. MgO calcined reductively catalyzes the reverse water gas shift (reaction 14), leading to CO generation. This results in reduced CO_2_ emissions below 820 °C, which means H_2_ increases above 820 °C [71,72,73]. However, reaction 14 was reported to occur above 1000 °C during iron oxides reduction [74].

(14) $${\mathrm{CO}}_{2}+{\;H}_{2\;}\overset{\mathrm{MgO}}{\to }\;\mathrm{CO}+H_{2}O\left(g\right)\;(\mathrm{reverse}\;\mathrm{water}\;\mathrm{gas}\;\mathrm{shift}\;\mathrm{reaction})$$

The reduction in CO_2_ emission is greater than the reduction in CH_4_ if no carbon deposition occurs during the dry reforming reaction (reaction 8) [42,75].

### 5.5. Methane Conversions

Resin to carbonates ratio during calcination governs the CO_2_/CH_4_ ratios. When resin/CaCO_3_ is equal to or less than one, CH_4_/CO_2_ emission is high (Figure 2). When resin/MgCO_3_ ratio is high, both CH_4_ and CO_2_ emissions are reduced by 94% (test 6, Figure 5). The presence of biomass during MgCO_3_ calcination results in higher CH_4_/CO_2_, while the CO_2_ is reduced up to 82% (Figure 4).

Increase in CH_4_ can be attributed to reaction 3. In total, 100% of the CO_2_ from MgCO_3_ calcination can be transformed to CH_4_ in the presence of H_2_ and the catalysts Co/Ca/CoO (reaction 15) [76].

CO_2_ + 4H_2_ → CH_4_ + 2 H_2_O; ∆H = −165 KJ mol^−1^ (15)

CH_4_ conversion (reduction in CH_4_) at high temp0eratures is ascribed to reactions 4, 8, and 9, leading to H_2_ generation (tests 5, 7, and 8). Reduction in CH_4_ (reaction 8) depends on CO_2_/CH_4_ ratios as well as the temperatures. A high CO_2_/CH_4_ ratio (reaction 8) results in high conversion of CH_4_, demonstrating the positive effect of CO_2_ as a soft oxidant at temperatures greater than 700 °C.

The dry reforming reaction (reaction 8) requires a cheap and pure source of CH_4_ and CO_2_. Pure CO_2_ is released during cement production from calcination of MgCO_3_ or CaCO_3_, and the CO_2_ from pyrolysis of biomass and the plastics ensure CO_2_ is greater than CH_4_ (reaction 2) (Table 4 and Table 5). It should be noted that more CO_2_ is released from MgCO_3_ (52%) compared to the CaCO_3_ (44%) stoichiometrically during calcination. Under the experimental conditions, MgCO_3_ calcination can result in sudden copious amounts of CO_2_ (calcination temperature~ 700 °C) compared to that from CaCO_3_ (calcination temperature ~900 °C). Increasing the amount of CH_4_ (from biomass and plastics) can increase H_2_ generation from reaction 4.

### 5.6. Hydrogen Generation

Figure 6 and Figure 7 show increased H_2_ and greatly reduced CO_2_ during the calcination of MgCO_3_ in the presence of plastics and biomass. It is anticipated CaCO_3_ follows a similar trend owing to its similar chemistry to MgCO_3_. This is attributed to reactions 4, 7, 8, and 9 directly contributing to increased H_2_ and syngas (H_2_ and CO).

### Co-Pyro-Gasification of Waste Plastics and Biomass vs. Individual Gasification of Wastes

Hydrogen enriched syngas production is attributed to several factors. Co-pyro-gasification of plastics and biomass blends increases the quality and the composition of syngas (H_2_/CO ratio) [66,77]. The present study showed the biomass and plastics blend enhanced the hydrogen generation while reducing CO_2_ and CH_4_ emissions. Using only plastics greatly reduced the CO_2_ emissions with negligible gen-eration of hydrogen; the biomass use only decreased CO_2_ emissions to an extent but fa-cilitated the generation of both H_2_ and CH_4_ (Figure 6 and Figure 7, Table 4 and Table 5). Co-pyro gasification of plastic wastes and biomass converts wastes predominantly to gas rather than to char and tar [77].

Increasing the CO_2_ promotes a high yield of syngas [73]. CaCO_3_ and MgCO_3_, plas-tic wastes and biomass, were the main sources of excess CO_2_ in the present study. When steam is present, the water gas shift reaction (reaction 6) shows reduction in CO and increase in H_2_ yields [72]. It should be noted that, in the present study, CO was not observed. If the water gas shift reaction 6 is not present, soot formation through me-thane cracking can occur (reaction 4).

MgO or CaO assisted reverse water gas shift reaction 14 results in increased H_2_ above 830 °C [69]. High H2 yield in reaction 8 is associated with high temperatures and low concentrations of CH4 (corresponding to increased conversion of MgCO_3_ to MgO and CaCO_3_ to CaO), i.e., high CO_2_/CH_4_ [30]. Reaction 4 favors higher H_2_ above 900 °C [73]. Excess water in methane reforming (reaction 9) results in complete oxidation of carbon and the exclusive production of H2 (reaction 16) instead of H_2_/CO.

CH_4_ + 2H_2_O → CO_2_ + 4H_2_ (∆H 298 K = +165 kJ/mol) (16)

### 5.7. Temperature Effects

In this stud, calcination of CaCO_3_ at 1450 °C (test 2) showed almost no CO_2_ content compared to calcination at 1250 °C (test 3) and increased methane in the absence of any external catalysts (Figure 2). The reductive H_2_ atmosphere can lower the calcination temperature by more than 150 °C compared to a non-reducing atmosphere [71]. The sudden spike in CH_4_ seen in Figure 2 may be attributed to reaction 11 from increased H_2_.

Increasing the gasification temperature of the biomass usually promotes syngas production while concurrently inhibiting the biochar production [78]. A slight decline in the syngas at temperature above 800 °C is ascribed to the reverse water gas shift reaction (reaction 14). Conditions for high H_2_ yield are discussed below.

High calcination temperatures increase H_2_ and CO contents, simultaneously decreasing the CO_2_ content by facilitating the hydrocarbons cracking (reaction 4) [47]. Reaction 4 favors higher H_2_ generation at temperatures above 900 °C [73]. CH_4_ formation is favored at low temperature and elevated pressure. There is a decrease in CO content at temperatures above 800 °C. High H_2_ yield is attributed to low amounts of CH_4_ in reaction 8 and high temperature when CO_2_/CH_4_ is high [60].

### 5.8. Char Formation

Char formation has immediate relevance to the endothermic carbon reforming reactions (reactions 8 and 9), which require catalysts. These catalysts also catalyze soot formation (reaction 4) [42] resulting in catalytic fouling, affecting the stability of the catalysts and increasing the costs of the dry reforming process (reaction 8), which hinders its commercialization. Reducing the char formation is important in carbon reforming reactions (reactions 8 and 9).

**Figure 8:**
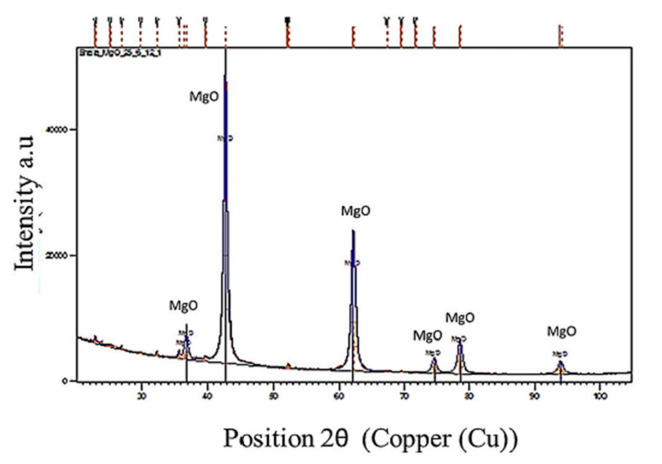
XRD of the calcined MgCO_3_·xH_2_O and the plastic blend at 1250 °C–Non-Soot forming.

In the present study, decreasing amounts of CH_4_ and CO_2_ and hydrogen generation during the calcination of MgCO_3_ confirmed the occurrence of carbon reforming reactions (reactions 8 and 9) without the aid of external catalysts. The XRD trace of the calcined MgCO_3_ at 1250 °C in the presence of plastics showed no carbon formation (Figure 8). This was attributed to the high temperatures (cement making temperatures) and the high amounts of pure CO_2_ generated from the calcination reactions (reaction 2) as well as from the co-pyro gasification of biomass and plastics.

Lignin present in biomass contributes to high amounts of char [79]. To suppress biochar production, it is necessary to increase the temperature and the heating rate, which can promote the syngas production [78]. Thermodynamic calculations indicate the required temperature to be 1035 °C for 50% CO_2_ conversion in reaction 8 without the catalyst. High CH_4_ and CO_2_ conversions at temperatures 700 °C require catalytic systems such as metal oxides, monometallic and bimetallic catalysts, and supported metal catalysts [80,81,82]. Steam reforming reactions (reactions 9 and 16) are favored at temperatures above 900 °C and 15–30 atm using nickel-based catalysts. However, carbon fouling of the catalysts is a serious issue.

Steam can eliminate the carbon formed as quickly as its formed. Alkali compounds improve the water gas reaction or the char reforming reaction (reaction 7) at temperatures above 700 °C [83,84]. Though reaction 5, the Boudouard reaction, can be a source of char formation, it does not occur above 700 °C [73]. When CO_2_/CH_4_ is high and temperature is above 700 °C, the coke deposition is diminished due to the oxidation reaction of CO_2_ with the surface carbon (reaction 5) [84].

Conversion of CO_2_ and CH_4_ is determined by the ratio of CO_2_/CH_4_ and the carbon or the soot formation [42]. Presenting CO_2_ to the catalytic dry reforming process (reaction 8) reduces the soot deposition. The CO_2_ from the calcination reactions of carbonates during cement making ensures CO_2_ is greater than CH_4_, thus reducing the carbon formation (Table 4 and Table 5). In the present study, more than 70% CO_2_ conversions were achieved without an external catalyst.

### 5.9. MgCO3–MgO and CaCO3–CaO Catalytic Systems Generated In Situ

The hydrogen generation in calcination reactions is governed by the steam (reactions 9 and 14) and the dry reforming reactions (reaction 8). The efficacy of these reactions relies on external catalytic systems, e.g., nickel-based catalysts. A 90% CO_2_ conversion was achieved for an MgO promoted catalytic system [75] promoting a partial reduction of CO_2_. In the present study, high conversions up to 99% were realized for both CH_4_ and CO_2_, accompanied by H_2_ generation without the use of external catalysts (Table 4). This was attributed to the MgCO_3_–MgO and the CaCO_3_–CaO systems acting as catalysts generated in situ.

Freshly prepared MgO and CaO on their own or in combination act as catalysts for the carbon conversion reactions [85]. The catalytic ability of CaO is better than MgO during the biomass conversions [86]. The characteristics that make MgCO_3_–MgO or CaCO_3_–CaO desirable catalysts for carbon reforming reactions include: Lewis basicity, mesoporosity, high reactivity and stability, small crystal size, high specific surface, high adsorption, and reduced carbon formation, promoting both steam forming and dry forming of CH_4_. Lewis bases considerably improve CO_2_ reforming of the CH_4_ reaction 8, resulting in values higher than the equilibrium values of H_2_. Freshly formed MgO from basic MgCO_3_ has a high specific surface, mesoporosity, low bulk density, low crystallite size, and nitrogen adsorption up to 100 cm^3^/g, making it catalytically active [50,51,52,87,88].

MgO calcined reductively catalyzes the reverse water gas shift reaction, leading to decreases in CO_2_ (reaction 14). The catalytic effect of CaO increases syngas production from mixed plastic wastes and from the halogenated plastics and the PVC fractions. Lime serves as a passage for fuel and gas and simultaneously binds halogen and other harmful pollutants [38,89]. CaO’s catalytic action prevents formation of dioxins and furan and tar containing cleavage products and oil at temperatures greater than 900 °C, hence facilitating the use of halogenated plastic waste streams. CaCO_3–_CaO suppresses the release of toxins such as C_6_H_6_ and HBr [90]. It was demonstrated that Portland cement making can effectively be treated as a plastic/biowaste and carbon conversion facility without the use of any costly external catalysts.

### 5.10. Syngas Production-Proposed Mechanism

It is proposed that one of the major reactions taking place during calcination in the presence of waste plastics and/or biomass is the reaction between CH_4_ from pyrolysis (reaction 3) and MgCO_3_ or CaCO_3_ to produce MgO or CaO and syngas (reactions 17 and 18). As calcination of carbonates progresses, the CO_2_ released reacts with CH_4_, resulting in increased amounts of H_2_ and CO (as MgO content increases, the amount of CH_4_ decreases, and the amounts of H_2_ and CO increase). Hence, it was concluded the MgCO_3_–MgO system or the CaCO_3_–CaO systems generated in situ effectively catalyzed the dry reforming reaction (reaction 8) without coke deposition (Figure 8).

MgCO_3_ + CH_4_ → MgO +2H_2_ +2CO (17)

CaCO_3_ + CH_4_ → CaO +2H_2_ +2CO (18)

However, CO (Figure 4, Figure 5, Figure 6 and Figure 7) was not detected in the present study. It is possible that high temperatures, composition of reactants, and CO_2_/CH_4_ could effectively suppress CO emissions. It was proposed that the catalytic actions of MgCO_3_–MgO and CaCO_3_–CaO systems not only catalyzed reactions 8 and 9 to produce H_2_/CO but also catalyzed the subsequent conversion of syngas to hydrogen and other, smaller hydrocarbon molecules, which could be building blocks to other useful fuels and chemicals. Composition of the reactants (e.g., MgCO_3_, resin, biomass) controlled the product gas distribution, e.g., as in selective production of H_2_ (test 7), or the mixed distribution of CO_2_:CH_4_:H_2_ in the product mixture in test 8.

## 6. Applications

Potential applications of the present study include extending similar strategies to more problematic materials, such as using halogenated waste materials in Sorel cements and Alinite clinker, and using silicones to replace sand, a costly commodity in Portland cement clinkers, and feedstock recycling of tires as sources of both plastics and biomass in cement making to combat high carbon footprint.

### 6.1. Decarbonising Sorel Cements and Alinite Clinker Using Halogenated Waste Plastics

Developing environmentally safe processes to handle halogenated plastic wastes is vital due to stringent environmental regulations. CaCO_3_ and MgCO_3_ inhibit the release of toxins such as C_6_H_6_, HBr, and dioxins, enhancing the pyrolysis process [90,91]. Hence, cement making is an ideal platform to repurpose halogenated plastic wastes.

Alinite clinkers utilize chlorine containing wastes, e.g., PVC [92]. Alinites is produced at 1150 °C, reducing the clinker formation temperature by 400–500 °C [93] with the potential to convert halogenated plastic wastes into hydraulic setting cements [94]. Heating the mixture of PVC, CaO, or Ca(OH)_2_ and Ni(OH)_2_ to 500 °C can fix CO_2_ and dechlorinate PVC, producing calcium hydroxide chloride (CaOHCl), CaCO_3_, and hydrogen (reactions 19 and 20). During this process, up to 90% H_2_ is released off as free gas [89].

2CO_2_ + 2CaO → 2CaCO_3_ (19)

CaO + HCl → CaClOH(20)

Introducing PVC during calcination of dolomite or limestone at temperatures above 900 °C aids H_2_ production without an external catalyst [69]. Application of PVC in Sorel cements (non-hydraulic cements) can follow similar reactions (reactions 19 and 20) at lower temperatures (750–800 °C) [17,94].

### 6.2. Silicones for Eco-Efficient Clinker Production

Concrete and cement clinker production use a significant amount of sand, the world’s second most consumed natural resource [95]. Silicones can replace high pure silica and coke in ferrosilicon production at cement making temperatures [61]. Silicone polymers possess organic and inorganic moieties with valuable resources such as silica, methane, carbon, and hydrogen. The organic moiety of silicones can reduce the emissions, while the inorganic moiety contributes silica. If co-pyro-gasified in the presence of biomass, the carbon emitted can be converted to hydrogen.

SiO_2_ from silicones can better replace the sand in cement clinkers in addition to offering similar emission and energy benefits derived from the waste plastics. Use of virgin silicones, siloxanes, and silanes in energy enabling technologies and as energy and materials results in energy savings and greenhouse gas (GHG) emission reductions. The CO_2_ emission cuts realized in Japan, North America, and Europe using virgin silicone products amount to ~54 Mt/y [96].

**Figure 9:**
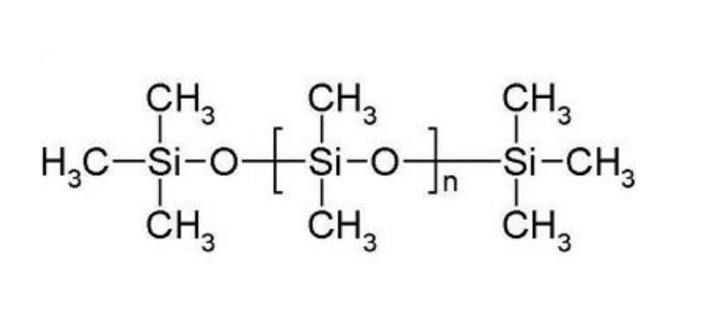
Structure of a linear silicone polymer (polydimethylsiloxane).

A pathway for the direct production of clinker (calcium silicates) from calcite and waste silicones to eliminate the use of silica is shown in reaction 21. This reaction demonstrates reduction in CO_2_ and energy consumption and simultaneous production of syngas and H_2_. Waste or virgin silicones can replace silica in reaction 1. CaCO_3_ calcined in the presence of silicones (polydimethylsiloxane (C_2_H_6_OSi) (Figure 9)) at cement making temperatures can directly produce clinker (CaSiO_5_) and syngas with a great reduction in CO_2_ emissions (reaction 21) while simultaneously facilitating silicone waste management.

CaCO_3_ + (C_2_H_6_OSi)n → CaSiO_5_ + CO + H_2_ (21)

### 6.3. Tires as Source of Both Plastic and Biomass

The present study indicates the potential use of tires as a chemical feedstock to gain the benefits of emission and energy as well as to use the rubber ash generated in situ during the pyrolysis/gasification as sand replacement in the cement system. Scientists are working on ways to replace sand in concrete with other materials, e.g., rubber tire ash. Rubber tires that comprise both synthetic rubbers (plastics) and natural rubbers (carbon neutral biomass) can be the ideal candidates to reduce the GHG emissions (CO_2_ and CH_4_) and to generate H_2_ in cement making, as proposed in the present study.

It should be noted that ash content from biomass and waste plastics is negligible and is unlikely to alter the material properties (ash content from plastics and wood—LDPE, HDPE, PP, and PVC—less than 0.05%; wood 0.45%; rubber tires 5.7%; and coke/coal 18.4%) [97].

### 6.4. Other Industrial Applications

This study has relevance not only to cement industries but also to iron and steel industries (where CaO and MgO are used as fluxes) in regard to dead burned magnesia production, carbothermic reduction of magnesium, carbon conversions, waste valorization, and emission and energy reduction while supporting the hydrogen economy and the generation of precursors for new materials. Use of plastics and biowastes can result in considerable reductions by about 200 °C in reaction temperatures (~1600 °C) during dead burned magnesia (DBM), fused magnesia (FM) production, and carbothermic reduction of Mg. Dead burned magnesia (DBM) currently makes up the largest portion of produced magnesia intermediate products, and there is growing demand and market share for FM [98].

## 7. Conclusions

IEA’s Sustainable Development Scenario (SDS) aim is to stay below 1.5 °C global warming, by adopting carbon mitigation strategies in cement sector. The findings of present study on decarbonsing the cement production using waste streams as chemical feedstock, and to simultaneously convert the CO_2_ produced during cement production to clean energy, are most relevant to the IEA’s SDS aim. The cement sector as a potential waste plastics/rubber tires treatment facility to simultaneously meet the emission targets, convert the GHG emissions to hydrogen, and maximize the recovery of resources present in waste materials, e.g., Si, H, CH_4_, and C, was discussed.

The study focused on developing Novacem-like low carbon cements and decarbonizing Portland cements. Use of waste plastics and biomass as chemical feedstock (co-pyro-gasification) to reduce the carbon footprint in the calcination step of cement making was demonstrated, which was never reported before. It should be noted the use of wastes as fuel in cement making was not considered in this study. Therefore, emission and energy benefits reported in this study were in addition to the benefits from using the wastes as fuel. Up to 99% reduction in GHG in Portland cement and Novacem-like cements production was established in this study.

The effects of temperature, the ratio of the plastics: biomass: carbonates in controlling GHG emissions, H_2_ production, and catalytic ability, and carbon fouling of the calcine intermediates are examined. High temperatures and high plastic content favored suppression of CO_2_ more than 95%, whereas biomass contributed to less suppression, i.e., up to 82% reduction in CO_2_ but up to 230% increase in hydrogen. A higher resin content than the biomass during calcination resulted in CO_2_ reduction up to ~99% and CH_4_ reduction up to ~97%, accompanied by 360% increase in H_2_ compared to the expected value. A higher biomass content than the resin during calcination resulted in 76% reduction in CO_2_ and ~63% reduction in CH_4_ but 4684% increase in H_2_ compared to the expected value. When CO_2_/CH_4_ were high and the temperature was above 700 °C, the coke deposition was diminished, thus preventing the carbon fouling of the catalytic calcine intermediates. Increasing the gasification temperature of the biomass also suppressed biochar formation. It was concluded that the catalytic actions of MgCO_3_–MgO and CaCO_3_–CaO systems not only catalyzed reactions 8 and 9, the carbon conversion reactions to produce H_2_/CO, but could catalyze subsequent conversion of syngas to hydrogen and other smaller hydrocarbon molecules as well.

Use of mixed plastics, including halogenated plastics, silicones, and biomass from the waste inventory as chemical feedstock in cement making was examined. CaCO_3_ minimizes the negative impacts of dioxins and toxic emissions from halogenated waste plastics during feedstock recycling and syngas production. The strategies presented in the present study can be applied to Alinite clinkers and Sorel cements production using halogenated plastic wastes with similar emissions and energy benefits.

Recommendations for direct clinker production from silicone/silicone wastes (as sand replacement), solid residues from tires (ash), and silicones (silica) from rubber tires or silicone polymers used as the feedstock can offer emission and energy benefits. They can replace sand in direct production of a cement clinker (CaSiO_5_).

## Figures and Tables

**Table 1:** Alternative cements for CO_2_ reduction [9].

Name	Type	Raw Material	Process Temperature	CO_2_ Reduction
Geopolymer	Alkali activated materials	Fly ash, Al/Si wastes, alkaline solutions	Ambient	Approx. 70%
Sulfolauminate cement	-	Limestone, gypsum, bauxite, sand/clay	1200–1300 °C	30–40%
Magnesia Binder (Novacem)	Magnesium oxide	Magnesium silicates	200 °C (180 bar) + 700 °C	greater than 100%
Magnesia Binder (TechEco)	Magnesium oxide + OPC + fly ash	MgCO_3_	<450 °C (Tec-Kiln)	greater than 100%
Celitement (KIT)	Calcium silicate hydrate	As OPC (Ca/Si ratio 1–2)	150–200 °C (hydrothermal)	Approx. 50%
Carbonatable Calcium Silicate cement (Solidia)	Calcium silicate (wollastonite)	As OPC for cement	1200 °C	Approx. 70%

**Table 2:** Net emissions factors for plastics for different materials management options [27,28].

GHG Emissions (kgCO_2_(e)/t Mixed Plastic)					
	Input Materials	Transport	Processing	Displacement Savings *	Net Emissions
Landfill	0.0	15.1	55.7	0.0	70.8
Incineration	0.0	15.1	2408.0	−565.5	1857.6
Pyrolysis	13.0	197.2	55.6	−425.5	−159.7
Gasification with MTG (methanol-to-gasoline process)	153.7	153.7	995.5	−261.7	1041.2
Gasification with F–T (Fischer–Tropsch process)	153.7	139.3	285.2	−147.1	431.1
Gasification with bio (gasification with biological conversion of syngas to ethanol)	153.7	187.7	1217.1	−454.9	1103.6
Catalytic depolymerization	16	197.5	51.0	−397.4	−132.8

* The avoided greenhouse gas (GHG) emissions associated with the displacement, where reuse occurs, and other product manufacture is displaced.

**Table 3:** Proximate and ultimate analysis of sawdust and plastic wastes.

	Pinus Radiata	Plastic Waste
Proximate analysis		
Ash/%	0.3	4.6
Volatile matter/%	87.5	91
Fixed carbon/%	12.2	3.2
Moisture		1.2
Ultimate analysis		
Carbon/%	50.1	69.8
Hydrogen/%	6.07	11
Nitrogen/%	0.21	0.5
Oxygen/%	43.2	13.7
Total sulfur/%	0.08	

**Table 4:** Summary of results involving isothermal calcination experiments using CaCO_3_ or MgCO_3_·xH_2_O.

Mass of Sample	Mass of Resin and/or Biomass, mg	Summary of Off-Gas Content	Figure No	Test No., Table No.
CaCO_3_ 2.36 g	0	54% CO_2_	2	1, 1450 °C
CaCO_3_ 2.36 g	Resin, 2.37 g	99.9% reduction in CO_2_ CH_4_ = 6.1% CO_2_/CH_4_ = 0.002	2	2, 1450 °C
CaCO_3_ 2.36 g	Resin, 2.06 g	88% reduction in CO_2_. CH_4_ = 4.8% CO_2_/CH_4_ = 1.7	2	3, 1250 °C
MgCO_3_·xH_2_O 11.39 mg	0	66.5% CO_2_;	3	Test 4, Table 4, 1000 °C
MgCO_3_·xH_2_O11.37 mg	Biomass, 28.99 mg	82% reduction in CO_2_ when CO_2_/CH_4_~1084. Substantial increase in CH_4_. Increase in hydrogen~230% and other hydrocarbons.	4	Test 5, Table 4, 1000 °C
MgCO_3_·xH_2_O 5.83 mg	Resin, 21.3 mg	~95% reduction in both CO_2_ and CH_4_ accompanied by negligible amounts of H_2_ when CO_2_/CH_4_ was greater than 10	5	Test 6, Table 4, 1000 °C
MgCO_3_·xH_2_O 5.7 mg	Resin + biomass, 10.32 + 7.17 mg	Reduction in CO_2_ (~99%) and in CH_4_ (~97%); 360% increase in H_2_ greater than expected when CO_2_/CH_4_ was greater than 10. Note: resin content about twice the MgCO_3_ content; biomass content close to the MgCO_3_ content	6	Test 7, Table 4, 1000 °C
MgCO_3_·xH_2_O6.5 mg	Resin + biomass, 7.72 + 8.09 mg	~76% reduction in CO_2_ and ~63% reduction in CH_4_. Considerable increase in H_2_ (4684%) when CO_2_/CH_4_ was ~24. Resin amount about three quarters that used in test 7; approximately equal amounts of resin, MgCO_3_, and biomass	7	Test 8, Table 4, 1000 °C

Note: In MgCO_3_ tests, the amounts of resin + biomass was greater than MgCO_3_. All tests were at 1000 °C.

**Table 5:** Cumulative gas compositions determined by gas chromatography from isothermal calcination reactions of calcium carbonate and magnesium carbonate with various ratios of biomass/ resin (plastics).

Total Sample Mass	Test No.	Cumulative Gas Composition y%				
		CH_4_	CO_2_	C_2_H_4_	C_2_H_6_	H_2_
2.36 g	1		54			
4.73 g	2	6.05	0.017			
4.42 g	3	4.79	8.2			
11.39 mg	4		66.46			
40.46 mg	5	2.12	13.87	0.53	0.24	0.33
26.58 mg	6	0.21	1.85	0.02	0.01	0.07
23.19 mg	7	0.049	0.05	0.01	0	1.15
22.31 mg	8	0.67	10.27	1.03	0.18	9.09

## Data Availability

Not Applicable.

## Nomenclature

International Energy Agency (IEA)	
Sustainable Development Scenario (SDS)	
Portland cement	
Novacem-like cements	
Alinite cements	
Sorel cements	
Feedstock recycling	
Calcite (limestone, CaCO_3_)	
Greenhouse gas (GHG)	
Lime (CaO)	
Magnesite (MgCO_3_)	
Magnesia (MgO)	
Gas chromatographic analyzer (GC)	
Mixed/halogenated plastic wastes	
Pinus radiata (biomass)	
Polyvinyl chloride (PVC)	
Silicones (polydimethylsiloxane).	
Tire	
MgCO_3_–MgO and CaCO_3_–CaO: catalytic calcine intermediates	
Carbon conversions (dry reforming and steam reforming reactions)	
Water gas reactions	
Water gas shift/reverse water gas shift reactions	
Boudouard reaction	
Syngas	
**Variables:**	
Temperature	
Sample weight:	
	Calcium carbonate
	Magnesium carbonate
	Plastics (resin)
	Biomass
CO_2_ reduction	
CH_4_ reduction	
H_2_ production	
Char formation/suppression	
