# Correction: Devasahayam, S. Decarbonising the Portland and Other Cements—Via Simultaneous Feedstock Recycling and Carbon Conversions Sans External Catalysts. *Polymers* 2021, *13*, 2462

**DOI:** 10.3390/polym14020281

**Published:** 2022-01-11

**Authors:** Sheila Devasahayam

**Affiliations:** Department of Chemical Engineering, Faculty of Science and Engineering, Monash University, Melbourne 3800, Australia; sheiladevasahayam@gmail.com

The author wishes to make the following correction to the above paper: [1]. In the original version of the published article, there is a mistake in Equation (16) on Page 14. It should be:

CH_4_ + 2H_2_O → CO_2_ + 4H_2_ (∆H 298 K = +165 kJ/mol) (16)

The author apologises for any inconvenience caused to the readers by these changes. These changes have no material impact on the conclusions of our paper. The original article has been updated.
